# Factors Correlated With Hepatitis C and B Virus Infections Among Injecting Drug Users in Tehran, IR Iran

**DOI:** 10.5812/kowsar.1735143X.806

**Published:** 2012-01-20

**Authors:** Masoumeh Amin-Esmaeili, Afarin Rahimi-Movaghar, Emran M. Razaghi, Ahmad Reza Baghestani, Siavash Jafari

**Affiliations:** 1 Iranian Research Center for HIV/AIDS, Tehran University of Medical Sciences, Tehran, IR Iran; 2 Psychiatry Department, Tehran University of Medical Sciences, Tehran, IR Iran; 3 Islamic Azad University, South Tehran Branch, Tehran, IR Iran; 4 Faculty of Medicine, School of Population and Public Health, The University of British Columbia, Vancouver, Canada

**Keywords:** Hepatitis Viruses, Prevalence, Risk Factors, Risk Behavior, Iran

## Abstract

**Background:**

In Iran, the number of injecting drug users (IDUs) has increased in recent years. The rates of hepatitis C virus (HCV) and hepatitis B virus (HBV) infections among IDUs are reportedly high.

**Objectives:**

The purpose of this study was to assess factors correlated with HCV and HBV infections among IDUs in Tehran.

**Patients and Methods:**

A cross-sectional study included 899 IDUs recruited from the community, drug treatment centers, and drop-in-centers. The study involved interviews conducted using an adapted version of the WHO Drug Injection Study Phase II (Version 2b) questionnaire and blood testing for the HCV antibody, hepatitis B surface antigen, and hepatitis B core antibody. A logistic regression model was used to identify independent factors correlated with HCV and HBV infections.

**Results:**

HCV infection was found to be primarily associated with female gender [odds ratio (OR) 5.0, 95% confidence interval (CI) 2.0-10.0)], unmarried status (OR 2.9, 95% CI 1.9-4.4), drug use for more than 10 years (OR 2.7, 95% CI 1.8-3.9), drug injection frequency of more than once per day (OR 2.6, 95% CI 1.6-4.2), history of imprisonment (OR 2.5, 95% CI 1.6-4.0)], and a history of shared injection needles in prison (OR 2.3, 95% CI 1.5-3.6). HBV infection was mainly correlated with a history of imprisonment (OR 1.9, 95% CI 1.4-2.7) and drug use for more than 10 years (OR 1.4, 95% CI 1.1-1.9).

**Conclusions:**

Because a considerable number of IDUs in Iran are receiving reduction services, tailoring services for prevention of hepatitis infection are necessary.

## 1. Background

For decades, Iran was facing a high rate of opium use, as a producer country. Thirty years after total eradication of opium poppy cultivation, Iran is experiencing a rapid shift towards more severe patterns of drug use. In 1999, it was estimated that 166,000 injecting drug users (IDUs) live in Iran [1]. Currently, it is estimated that there are more than 260,000 IDUs in the country [2]. To address this sharp increase in injection drug use and its health-related consequences, harm-reduction initiatives were started in 2002 and have expanded since 2005. Hepatitis C virus (HCV) is a major cause of chronic liver disease worldwide and a potential contributor to morbidity and mortality [3]. In Iran, the prevalence of HCV infection in the general population has been estimated to be less than 1% [4]; this is significantly less than the estimated prevalence in the world population (3%) [5]. Since initiation of routine screening for HCV in donated blood, transfusions have virtually been eliminated as a source of HCV transmission. Therefore, most newly acquired cases of hepatitis C are related to injecting drug use, mainly due to unsafe injection practices [6][7][8]. The prevalence of hepatitis B virus (HBV) infection in the general population of Iran ranges from 1.7% to 5% [9], which is in an intermediate range of endemicity [10]. In a review, Custer et al. found that the prevalence of chronic HBV infection in the general population was highly variable, ranging from greater than 10% in some Asian and Western Pacific countries to less than 0.5% in the United States and northern European countries [11]. Additionally, injecting drug use was identified as a risk factor for hepatitis B in many countries [11].

## 2. Objectives

High rates of HCV and HBV infections among IDUs have been reported in Iran [12][13][14]. Few studies have evaluated the risk factors related to these rates. This study is a part of a larger study examining the characteristics, risk behaviors, and risk factors of hepatitis C, hepatitis B, and human immunodeficiency virus (HIV) infections among IDUs in Tehran, the capital city of Iran. This paper presents the results related to the prevalence of HCV and HBV infections and the correlating factors in this population.

## 3. Patients and Methods

### 3.1. Population and Field Work

This cross-sectional study was conducted between June 2006 and March 2007 and involved 904 current IDUs selected from drug treatment centers and the community. Drug users were included in the study if they had a history of injecting drug use over the 2-month period prior to the study and consented to participate in the interviews. Using purposive sampling with ethnographic observations in public places, peer referrals, and assessments of snowballing effects, a community sample population was selected from five areas of Tehran with high rates of drug use. Another sample population was selected from three drug-treatment centers and two drop-in-centers (DICs) located in different, well-known areas of Tehran with high rates of drug use. Treatment centers provide methadone maintenance treatment (MMT), and DICs mainly provide other types of harm-reduction interventions, including needle and syringe programs (NSP) and harm-reduction education. Cases were selected based on consecutive admissions to the centers during the study time period. We used the questionnaire of the "WHO Drug Injection Study Phase II (Version 2b)" for data collection. Three psychiatrists adapted the questionnaire according to the situation in Iran, which was followed by a pretest assessment on a sample of IDUs. The questionnaire included assessments of socio-demographic characteristics, drug use and injecting drug use practices, sexual risk behaviors, knowledge regarding hepatitis infection, and service use.

Fieldwork, including interviews and blood sampling, was carried out by experienced drug therapists and outreach workers after a short training course that included questionnaire training and instructions on transferring blood samples to the laboratory. There was no monetary incentive for participation in this study. However, the use of free-of-charge health services, including drug treatment, was offered as an incentive.

### 3.2. Blood Sample

Blood samples were tested for the HCV antibody (anti-HCV ELISA, DRG Co., Germany), hepatitis B surface antigen (HBsAg IEMA WELL, Radim, Italy), and hepatitis B core antibody (anti-HBc EIA WELL, Radim, Italy). A positive result either for HBsAg or anti-HBc was considered to indicate "past or current HBV infection." All positive and borderline-positive samples for HBsAg, anti-HBc, and anti-HCV were retested using the same method. We excluded samples that repeatedly showed borderline-positive results. All tests were conducted at the Keyvan Laboratory.

### 3.3. Statistical Analysis

Statistical analyses were performed using SPSS for Windows (version 16.0, 2007; SPSS Inc., Chicago, IL, USA). Univariate analysis was conducted using chi-square test for binomial variables. All variables were included in the multivariable analysis. A multivariate logistic regression model using a forward conditional method was used to identify independent correlates of HCV and HBV infections. Associations were assessed using the odds ratios and 95% confidence intervals, and adjusted odds ratios were determined through multivariate analysis.

### 3.4. Ethical Considerations

The research protocol was approved by the ethics committee of Tehran University of Medical Sciences in Iran. Participation in the study was on a voluntary basis, and informed consent was obtained for the interview as well as for collecting blood samples. All efforts were made to guarantee privacy and confidentiality during the interviews. A de-linked method for testing was carried out to ensure confidentiality of the results. An identification code, which was used for laboratory results as well, was included on each questionnaire. Participants were ensured that non-participation in the study would not affect their treatment or harm-reduction service utilization.

## 4. Results

A total of 904 IDUs were enrolled in the study. Most of the participants (95.8%) were male. Participant ages ranged from 16 to 65 years, with a mean age [standard deviation (SD)] of 33.9 (9.4) years. The mean (SD) years of education was 7.7 (3.5) years. Most subjects were unmarried (70.8%) and unemployed (64.1%). Socio-demographic profiles of participants recruited from drug treatment centers, DICs, and the community are presented in Table 1. The mean (SD) age at first drug use was 19.7 (4.0) years. The main injected drug used within the last 6 months was heroin (81.3%), and the mean (SD) duration of drug injection was 8.4 (7.6) years. Most participants (76.6%) reported injecting with used injection equipment, including needles and syringes, or sharing other injection paraphernalia within the last six months. Among the participants, 70.9% had a history of imprisonment, and 21.5% of these had a history of sharing injection equipment in prison.

**Table 1:** Socio-Demographic Profile of the Study Sample in Three Different Settings

	**Drug Treatment Center (n = 158)**	**DICs a (n = 290)**	**Community (n = 446)**
Gender			
Female	1	1	1
Male	20	20	25
Age, y, mean ± SD	34.2 ± 9.4	34.2 ± 9.8	33.5 ± 9.2
Full-time education, y, mean ± SD	8.0 ± 3.7	8.2 ± 3.3	7.3 ± 3.5
Single; widowed; or separated, %	47.8	71.2	78.7
Homeless, %	5.8	30.1	56.4
Living alone, %	14.5	34.7	56.2
Unemployed, %	26.9	67.2	75.6
History of imprisonment, %	38.6	75.9	78.8

^a^ Abbreviation: DICs, drop-in centers

With regard to sexual behavior, 36.4% of the participants reported an extramarital relationship (either heterosexual or homosexual) within the last 6 months, and of these, 80% had a history of intercourse without protection during the same period. An extramarital relationship without protection was defined as "high-risk sexual behavior." Five of the interviewees refused to participate in blood testing. In the HCV antibody testing, four samples showed repeated borderline results. Of the remaining 895 participants, 34.5% (CI 31.4-37.7%) tested positive for HCV. In univariate analysis, several variables were identified as factors correlated with transmission of hepatitis C among the participants. Recruitment from DICs and the community, older age, unmarried status, homelessness and living alone, history of imprisonment and unsafe sharing practices in prison, initiation of drug use at a young age, longer duration of drug use and duration of injecting drug use, more frequent injecting drug use, history of overdose, and recent use of harm-reduction services were associated with a higher prevalence of HCV infection (Table 2).

Multivariate logistic regression analysis revealed that the following independent factors were associated with HCV infection: recruitment from DICs and the community, female gender, unmarried status, longer duration of drug use, injection frequency of more than once per day, history of imprisonment and history of shared injection in prison, and use of a harm-reduction service during the past six months. The analysis also showed that sharing injection paraphernalia during the past six months was reversely associated with HCV infection (Table 2). Further analysis showed that among participants who had used harm-reduction services in the past six months, the rate of recent sharing behavior was less than those who had not used the services (73.2% vs. 79.9%, P < 0.02(. HBV testing showed borderline results or insufficient samples in 35 (3.5%) cases, which were excluded from the analysis. Of the 864 remaining subjects, 40 cases showing a weak positive result for HBsAg and negative results for anti-HBc were regarded as negative cases. HbsAg was detected in 24.7% and anti-HBc in 29.1% of the 864 cases. Overall, 46.1% of cases showed either past or current HBV infection. Univariate analysis revealed older age, history of imprisonment, longer duration and more frequent drug use, history of drug overdose, and recent drug-treatment service use as risk factors for HBV infection among IDUs. Multivariate logistic regression analysis showed that history of imprisonment and a longer duration of drug use were independently correlated with HBV infection (Table 2).

**Table 2:** Univariate and Multivariate Analysis of Risk Factors for HCV and HBV Infection

	**HCV Infection**				**HBV Infection**			
	**Patients, No.**	**HCV (+), No. (%)**	**OR a (95% CI)**	**AOR a (95% CI)**	**Patients, No.**	**HBV (+), No. (%)**	**OR (95% CI) a**	**AOR (95% CI)**
Recruitment setting								
Drug treatment center	158	17 (10.8)	1	1	152	74 (48.7)	1	-
Drop-in centers	290	132 (45.5)	7.0 (4.0–12.1)	5.7 (2.8–11.2)	282	125 (44.3)	0.8 (0.6–1.2)	-
Community	446	160 (35.9)	4.6 (2.7–8.0)	3.3 (1.7–6.4)	429	218 (46.2)	0.9 (0.6–1.3)	-
Gender								
Female	36	16 (44.4)	1	1	37	13 (35.1)	1	-
Male	859	293 (34.1)	0.6 (0.3–1.3)	0.2 (0.1–0.5)	827	385 (46.6)	1.6 (0.8–3.2)	-
Age, y								
≤ 35	561	161 (28.7)	1	-	545	234 (42.9)	1	-
> 35	332	146 (44)	2.0 (1.5–2.6)	-	317	163 (51.4)	1.4 (1.1–2.0)	-
Education								
≤ Grade 8	475	168 (35.4)	1	-	456	224 (49.1)	1	-
> Grade 8	414	138 (33.3)	0.9 (0.7–1.2)	-	402	173 (43.0)	0.8 (0.6–1.03)	-
Marital status								
Married and living with a partner	264	62 (23.5)	1	1	252	111 (44.0)	1	-
Single, widowed, or separated	631	247 (39.1)	2.1 (1.5–2.9)	2.9 (1.9–4.4)	612	287 (46.9)	1.1 (0.8–1.5)	-
Main place of residence during last 6 months								
Have a place to live	541	174 (32.2)	1	-	521	247 (47.4)	1	-
Homeless	343	133 (38.8)	1.3 (1.01–1.8)	-	334	149 (44.6)	0.9 (0.7–1.2)	-
Living alone								
No	520	166 (31.9)	1	1	501	244 (48.7)	1	-
Yes	368	141 (38.3)	1.3 (1.0–1.8)	0.7 (0.4–1.0)	356	152 (42.7)	0.8 (0.6–1.03)	-
Current employment status								
Employed	317	97 (30.6)	1	-	305		1	1
Unemployed	566	207 (36.6)	1.31 (0.98–1.8)	-	547	241 (44.1)	0.8 (0.6–1.04)	0.7 (0.5–0.9)
History of imprisonment								
No	259	38 (14.7)	1	1	252	94 (37.3)	1	1
Yes	631	268 (42.5)	4.3 (2.9–6.3)	2.5 (1.6–4.0)	607	302 (49.8)	1.7 (1.2–2.2)	1.9 (1.4–2.7)
Age of first drug use, y								
≤ 20	583	217 (37.2)	1	-	560	269 (48.0)	1	-
> 20	311	92 (29.6)	0.7 (0.5–0.95)	-	303	128 (42.2)	0.8 (0.6–1.05)	-
Duration of drug use, y								
≤ 10	344	75 (21.8)	1	1	331	128 (38.7)	1	1
> 10	542	231 (42.6)	2.7 (1.96–3.6)	2.7 (1.8–3.9)	525	265 (50.5)	1.6 (1.2–2.1)	1.4 (1.1–1.9)
Duration of injecting drug use, y								
≤ 2	223	41 (18.4)	1	-	218	88 (40.4)	1	-
> 2	660	264 (40)	2.96 (2.04–4.3)	-	635	304 (47.9)	1.4 (0.99–1.9)	-
Frequency of injection during last 6 mounths								
≤ once daily	210	42 (20)	1	1	209	80 (45.5)	1	-
> once daily	684	266 (38.9)	2.6 (1.8–3.96)	2.6 (1.6–4.2)	654	317 (48.5)	1.5 (1.1–2.1)	-
Sharing needle/syringes during last 6 mounths								
No	325	104 (32)	1	-	315	137 (43.5)	1	-
Yes	566	204 (36)	1.2 (0.9–1.6)	-	545	261 (47.9)	1.2 (0.9–1.6)	-
Any sharing behavior during last 6 mounths								
No	212	64 (30.2)	1	1	208	87 (41.8)	1	-
Yes	683	245 (35.9)	1.3 (0.9–1.8)	0.5 (0.3–0.8)	656	311 (47.4)	1.3 (0.9–1.7)	-
History of sharing injection in the prison								
No	759	236 (31.1)	1	1	735	330 (44.9)	1	-
Yes	136	73 (53.7)	2.57 (1.8–3.7)	2.3 (1.5–3.6)	129	68 (52.7)	1.4 (0.9–2.0)	-
Injection with pre-filled syringe during last 6 mounths								
No	693	241 (34.8)	1	-	665	300 (45.1)	1	-
Yes	200	68 (34)	0.97 (0.7–1.4)	-	197	98 (49.7)	1.2 (0.9–1.7)	-
High risk sexual behavior during last 6 mounths								
No	634	217 (34.2)	1	-	609	285 (46.8)	1	-
Yes	261	92 (35.2)	1.1 (0.8–1.4)	-	255	113 (44.3)	0.9 (0.7–1.2)	-
History of drug overdose								
Yes	394	163 (41.4)	1	-	480	199 (41.5)	1	-
No	493	141 (28.6)	1.8 (1.3–2.3)	-	376	195 (51.9)	1.5 (1.2–2.0)	-
Naming hepatitis C								
No	413	134 (32.4)	1	-	318	149 (46.9)	1	-
Yes	475	174 (36.6)	1.2 (0.9–1.6)	-	540	247 (45.7)	0.9 (0.7–1.3)	-
Used any harm reduction service during last 6 mounths								
No	432	129 (29.9)	1	1	414	195 (47.1)	1	-
Yes	463	180 (38.9)	1.5 (1.1–2.0)	1.5 (1.02–2.1)	449	203 (45.2)	0.9 (0.7–1.2)	-
Used any drug treatment service during last 6 mounths								
No	204	60 (29.4)	1	-	200	73 (36.5)	1	-
Yes	691	249 (36)	1.4 (0.96–1.9)	-	663	325 (48.9)	1.7 (1.2–2.3)	-

^a^ Abbreviations: AOR; adjusted odds ratio, CI; confidence interval, OR; odds ratio

## 5. Discussion

In this study, we investigated the factors correlated with the transmission of HCV and HBV among IDUs in Tehran, the capital city of Iran. This is the largest study evaluating HCV and HBV infections and their correlated factors among IDUs in Iran. Only one study, which was conducted in 2004, has assessed the factors correlated with HCV infection among 202 non-incarcerated male IDUs in Tehran [13].

### 5.1. Prevalence of Hepatitis C in IDUs

We found that the prevalence of HCV was 34.5% in the sample IDU population. Among studies with a minimum sample size of 100, there have been three studies examining non-incarcerated IDUs in Iran; in these studies, the prevalence of HCV infections was 11.2% [12], 30.5% [14], and 52% [13]. Among incarcerated IDUs, reported HCV prevalence rates are higher, ranging from 31.5% to 81.8% [14][15][16][17][18]. HCV prevalence among non-incarcerated IDUs has also been reported in neighboring countries. In Afghanistan, the prevalence of HCV is reportedly 36.6% in the capital city of Kabul [19]; Pakistan reported higher rates, ranging from 88% to 94.3% [20][21][22]. In a recent report, the HCV prevalence among IDUs in South and Southeast Asia ranged from 10 to 100% [23]. Our study showed a medium HCV prevalence rate among non-incarcerated IDUs.

### 5.2. Variables Correlated With Hepatitis C

Our findings indicate that HCV infection is associated with female gender, a history of imprisonment and shared injection in prison, drug injection frequency of more than once per day, drug use for more than 10 years, and unmarried status. A key finding of this study is the association between gender and the risk of hepatitis C transmission. A similar finding has been reported in England, in which female gender was identified as a risk factor for HCV infection [24]. Other studies have not identified such a relationship [25][26]. Several social and demographic factors may predispose female drug users to acquiring blood-borne infections. A lack of gender-specific services in communities, stigma, financial dependence on a partner, and partner pressure [27][28] have been identified in other studies in Iran as limiting factors for female drug users' access to and utilization of treatment and prevention resources. Involvement in high-risk behaviors such as sex-for-money and sex-for-drugs have been reported as well. Similar to our study, other studies have reported imprisonment as a major risk factor for hepatitis C transmission in Iran and around the world. In Iran, the duration of incarceration (> 1 year) was previously identified as a risk factor [13]. These findings are consistent with studies conducted in Western countries that identified history of imprisonment, including duration and/or number of incarcerations, as factors associated with HCV seroconversion [29][30][31]. Our study indicated that approximately 70% of participants had a history of imprisonment, of which more than one-fifth shared needles while in prison. Since 2005, harm-reduction interventions inside Iranian prisons have been expanded; however, only one qualitative study has reported a significant decrease in injection drug use in one prison [32].

We found that drug injection frequency of more than once per day and drug use duration of more than 10 years were associated with high rates of HCV infection. Duration of injection drug use (> 10 years) has been reported as a risk factor in another study in Iran [13]. These findings are consistent with the results reported in other countries [22][25][29][33][34]. However, we found an HCV seroprevalence of 18.2% among IDUs who had been injecting drugs for less than two years. This finding indicates a more rapid rate of infection and suggests that infection occurs shortly after the initiation of injection practices. Other reports have also reported a rapid increase in HCV infection upon initiation of drug injection [35][36][37][38]. The high prevalence of hepatitis C infection among IDUs participating in high-risk behaviors such as needle sharing make unsafe injection the primary cause of hepatitis C transmission in developed countries [5][34]. Studies conducted in countries in this region, such as Pakistan [39] and Georgia [40], have reported unsafe injection to be the main cause of hepatitis C transmission among their populations. However, a meta-analysis of the factors correlated with HCV in China showed no significant difference between needle-sharing IDUs and non-needle-sharing IDUs [25]. In our study, sharing behavior during the last six months was reversely associated with HCV infection. This result, along with the finding of no relationship between HCV infection and knowledge of hepatitis C infection, suggests that the knowledge and behavior of IDUs with a long history of drug use and injecting drug use have improved. This also correlates with the finding that HCV infection is more prevalent among IDUs who used harm-reduction services during the last six months than among those who had not used the services.

In this study, no association was identified between recent high-risk sexual behavior and hepatitis C infection. Other studies also found HCV infection to be unrelated to sexual practices [26][33][35][40][41], confirming that hepatitis C among IDUs appears to be a consequence of repeated exposure to contaminated injection equipment.

### 5.3. Prevalence of Hepatitis B Among IDUs

The prevalence of past or current HBV infection was 46.1% in the study population. In another study examining incarcerated IDUs in Tehran, HBcAb was detected in 61.2% of the participants [42]. Among studies with a minimum sample size of 100, only one study examining non-incarcerated IDUs was conducted in Iran, which reported a positive HBsAg result in 6% of the cases [12]. The reported rates of IDUs in Kabul (6.5%) [19] and Karachi (7.5%) [20] are also relatively low. Injecting drug use is reportedly the primary risk behavior in new cases of hepatitis B in males in Egypt [43]. There are also reports of a greater than 50% rate of HBV infection among IDUs in other countries [40][44][45]. However, the high rate of HBV infection found in our study requires further investigation. Notably, in Iran, a national program for hepatitis B vaccination in newborns was begun in 1992; however, no specific vaccination plan for drug users has been established.

### 5.4. Variables Correlated With Hepatitis B

This is the second study that has explored factors correlated with HBV infection among IDUs in Iran. In our study, HBV infection was mainly correlated with a history of imprisonment and drug use for more than 10 years. These results are consistent with a report from Iran [46] and reports from other countries regarding risk factors of HBV infection in IDUs that have identified imprisonment [45][47] and the frequency [48] and duration of drug injection [45][49] as risk factors of HBV infection.

### 5.5. Limitations of the Study

Drug use is often a hidden behavior, and drug users are considered a hard-to-reach population. Therefore, selecting a representative sample of drug users in a geographical area is difficult. However, methods that are more representative are recommended, such as respondent-driven sampling or a hybrid sampling plan using several methods [50][51], as was used in our study. Additionally, sampling was conducted such that a higher number of IDUs in Tehran were involved in the study, and the results may not be generalized to the entire Iranian IDU population. Another limitation is that study participants were interviewed regarding their past circumstances and recall bias may be an issue. Additionally, respondents may have been under influence of drugs at the time of interview and may not have provided precise data. This study had a cross-sectional design, and the temporal relationship between correlated factors and HCV and HBV seropositivity cannot be proven.

Blood-borne infections pose a serious threat to the health of injecting drug users. Factors related to hepatitis B and C virus infections appear to be similar among IDUs. The findings explain the reasons behind the high rate of co-infections found in our study and explained elsewhere [52]. Since 2005, there have been continuous efforts to increase coverage of harm-reduction interventions in Iran. The intermediate prevalence of hepatitis C infection and the high rate of hepatitis B infection observed in our study highlight the importance of additional studies on the long-term effectiveness of harm-reduction interventions. Reports from other countries have also demonstrated that HIV preventive measures for IDUs are not always effective in controlling viral hepatitis [53]. Additionally, prison is a known predisposing environment for HIV transmission in Iran. Our study revealed the importance of prisons in acquiring HCV and HBV infection, as well. However, as a considerable number of IDUs in Iran are covered under harm-reduction programs, either in the community or in prison [54], these results present a good opportunity to tailor services for hepatitis patients to decrease exposure to contaminated blood as well as risk reduction in sexual behaviors. We also suggest primary prevention of initiating injection drug use in addition to a provision for hepatitis B vaccination and HCV and HBV infection testing for IDUs.
